# Functional Results of Surgical Treatment of Bilateral Lesions of the Recurrent Laryngeal Nerve

**DOI:** 10.7759/cureus.91043

**Published:** 2025-08-26

**Authors:** Rajko Jovic, Ksenija Samac Tovilovic, Danijela Dragicevic, Jugoslav Gasic, Vanja Tovilovic, Ivan Sivcev, Mladen Bogdanovic

**Affiliations:** 1 Department of Otorhinolaryngology, University of Novi Sad, Faculty of Medicine in Novi Sad, Novi Sad, SRB; 2 Department of Otorhinolaryngology, University of Priština in Kosovska Mitrovica, Faculty of Medicine, Kosovska Mitrovica, SRB; 3 Department of Otorhinolaryngology and Head and Neck Surgery, University Clinical Centre of Vojvodina, Novi Sad, SRB

**Keywords:** arytenoidectomy, bilateral palsy, laterofixation, pulmonary function test, vocal cord

## Abstract

Background: Bilateral lesions of the recurrent laryngeal nerve require urgent deobstruction in acute conditions, and in chronic conditions, one of the surgical interventions is to widen the glottic space.

Objective: To indicate the short- and long-term functional effects of surgical deobstruction of the glottic space in bilateral lesions of the recurrent laryngeal nerve.

Methods: This study is a retrospective analysis of the treatment outcomes of 46 patients with bilateral lesions of recurrent laryngeal nerves, who were monitored using values of pulmonary function tests and visual analog scale scores of breathing preoperatively and one and six months postoperatively.

Results: Airflow parameters after endo-extralaryngeal laterofixation showed statistically significant improvement after one month of intervention in forced expiratory volume in one second (FEV1) (p = 0.0252), while forced vital capacity (FVC%) (p = 0.0835), peak expiratory flow (PEF%) (p = 0.0605), and peak inspiratory flow (PIF) (p = 0.0626) showed the limit of significance. The significance of values was lost after six months. Airflow parameters after posteromedial arytenoidectomy with transmuscular posterior cordectomy, mucosal preservation, and laterofixation (PMATMPCMPL) showed a statistically significant improvement in PEF (p = 0.0249) and PIF (p = 0.0476) one month after surgery. At six months, a statistically significant difference was observed in FEV1 (p = 0.0134), PEF (p = 0.0182), and PIF (p = 0.0173).

Conclusion: Airflow parameters after endo-extralaryngeal laterofixation are better one month after surgery, while the results of PMATMPCMPL are better six months after surgery.

## Introduction

Bilateral vocal fold paralysis in adduction, resulting from recurrent laryngeal nerve injury, is characterized by a paramedian position of the vocal folds, leading to inspiratory dyspnea due to significant glottic narrowing [1,2]. The most common cause of such injury is iatrogenic, typically occurring during neck surgeries (thyroid, parathyroid, thymus, esophagus surgeries, or carotid body paraganglioma removal) [3]. It is estimated that total thyroidectomy accounts for 80% of bilateral recurrent laryngeal nerve injuries, with the incidence of this complication noted in 1% of total thyroidectomies [3,4]. Symptoms usually manifest immediately after surgery, often during failed extubation attempts, necessitating repeated intubations [1]. Definitive diagnosis primarily relies on flexible fiberoptic laryngoscopy, performed in an awake patient to evaluate spontaneous and voluntary vocal fold movements [5].

Management of this condition involves one of the several described surgical techniques, all aiming to avoid tracheostomy, which, although reliable, remains a highly unpopular definitive method [1]. Laterofixation is one of the treatment options and represents a reversible procedure, wherein a translaryngeal introduction of a needle through an endo-extralaryngeal holder enables lateralization of one vocal fold. Due to its reversibility, this intervention can be performed immediately upon the onset of acute symptoms associated with bilateral vocal fold paralysis, without waiting for potential functional recovery [6]. Endoscopic arytenoidectomy is an irreversible procedure involving the endoscopic removal of the arytenoid cartilage to achieve a transverse enlargement of the glottic airway, thereby improving the inspiratory passage. It can be performed alone or in combination with vocal fold resection, in which case it is termed arytenoid cordectomy. This technique has shown favorable outcomes in terms of improved ventilation in patients with bilateral vocal fold paralysis. However, it also carries the risk of scar and granuloma formation, which may lead to airway narrowing and necessitate multiple surgical revisions [5]. According to the European consensus published this year, the main procedures in the care of patients with bilateral vocal cord paralysis can be divided into several categories with further subclassification. Main procedures are total arytenoidectomy, partial arytenoidectomy (subtotal, anteromedial, posteromedial, superomedial), posterior cordectomy (ligament and transmuscular posterior cordectomy and posterior ventriculocordectomy), and transverse cordotomy (posterior cordotomy, posterior ventriculocordectomy). Three different suffixes can be added to all arytenoid and vocal fold procedures, including "with mucosal preservation," "with laterofixation," and "combined procedure" [7].

The aim of the study is to indicate the short-term and long-term functional effects of surgical deobstruction of the glottic space in bilateral lesions of the recurrent laryngeal nerve using Lichtenberger laterofixation in acute and posteromedial arytenoidectomy with transmuscular posterior cordectomy, mucosal preservation, and laterofixation in permanent lesions of the recurrent laryngeal nerve.

## Materials and methods

A retrospective analysis of treatment outcomes of 46 patients with bilateral lesions of the recurrent laryngeal nerves was conducted. In addition to clinical monitoring and comparison of the condition before and after the operation, the values ​​of pulmonary function tests were also obtained.

The mean age of the patients was 52 years (minimum = 6 years, maximum = 79 years, SD = 12.4 years), of which two were men and 44 were women. All patients were operated on by the same operator, using the same technique, at the Otorhinolaryngology Clinic of the University Clinical Center of Vojvodina.

Twenty-two patients with fresh lesions (up to two months old) underwent Lichtenberger's endo-extralaryngeal laterofixation of the vocal fold under either jet ventilation or general anesthesia, using tube number 5 or 6. A monofilament 2-0 non-resorbable thread was used to place two threads at the level of the vocal process of the arytenoid, above and below the vocal fold, with the help of a Lichtenberger needle holder. Sutures were placed through a small incision on the skin of the neck, up to 1 cm under the skin. The subcutaneous tissue and infrahyoid muscles were separated, and then sutures were placed as close as possible to the thyroid cartilage. Antibiotics and corticosteroids were given for two days, and if necessary, anti-inflammatory and anti-edematous therapy was also provided.

For older lesions (at least six months after their onset) with no signs of laryngeal nerve recovery, posteromedial arytenoidectomy with transmuscular posterior cordectomy, mucosal preservation, and laterofixation using an endo-extralaryngeal approach, according to Lichtenberger's technique, was performed. There were 24 patients, 19 of whom had no prior intervention, and five patients in whom medialization occurred after laterofixation of the vocal cord according to Lichtenberger's technique. Under general anesthesia or jet ventilation, using cold steel instruments, posteromedial arytenoidectomy with transmuscular posterior cordectomy, mucosal preservation, and laterofixation was performed.

Resection of the vocal process of the arytenoid and submucosal removal of the posterior third of the vocal muscle were done. In cases of short vocal folds, half of the vocal muscle was removed. By carefully cutting the conus elasticus, the tension of the mucosa was reduced. With the Lichtenberger needle holder, two threads were placed around the mucosa to move it laterally, thus reducing the resulting defect. The threads remain on the skin for seven to 10 days. Postoperatively, antibiotics and corticosteroids were administered for two days, then anti-inflammatory therapy was given as needed.

The effect of both interventions was monitored anamnestically and clinically, with the assessment of the patient's subjective condition and local clinical findings. Pulmonary function tests were monitored only in patients in whom it was possible to compare the condition before one of the interventions, one month later, and six months later. Patients who did not undergo preoperative functional processing due to the severity of their clinical picture, the need for urgent intervention, or the existence of a tracheostomy were not included in that part of the study.

As functional processing involves a large number of parameters, the parameters that show the actual inspiratory airflow, including peak inspiratory flow (PIF; L/s), forced inspiratory flow at 50% (FIF50; L/s), expiratory airflow through the percentage of the ratio of the current and predicted value for forced vital capacity (FVC), forced expiratory volume in one second (FEV1), and peak expiratory flow (PEF), and the parameters that show the resistance in the airways through the percentage of the ratio of the current and predicted value for respiratory total resistance (Rtot) and reference values (Reff) are singled out. By processing these parameters, data that clearly indicated the state of air flow during inspiration, expiration, and resistance in the respiratory tract were considered.

Preoperatively and postoperatively, patients evaluated their breathing at rest and with effort on a visual analog scale. The score ranged from 1 to 10, with 1 being extremely difficult breathing and 10 being normal breathing [6].

The tested material has a coefficient of variation of less than 30, so the paired T-test was applied.

## Results

Bilateral lesions of the recurrent laryngeal nerve resulted from several etiologies: iatrogenic injury following total thyroidectomy in 41 (89.1%) patients; iatrogenic injury after carotid artery surgery in two (4.3%) patients; attempted suicide by slaughter in two (4.3%) patients; and an idiopathic cause in one (2.1%) patient.

The earliest referred patient with a bilateral lesion of the recurrent laryngeal nerves was eight days after the onset of paresis, directly from the intensive care unit, after multiple extubation attempts that ended with reintubation. The latest patient presented five years after the lesion with signs of difficulty breathing.

Observing the results of operative treatment one month after endo-extralaryngeal laterofixation and posteromedial arytenoidectomy with transmuscular posterior cordectomy, mucosal preservation, and laterofixation showed a statistically significant increase in FVC (preoperatively = 86.252, one month postoperatively = 93.6174, p = 0.0286), FEV1 (preoperatively = 56.7741, one month postoperatively = 77.0963, p = 0.0072), PEF (preoperatively = 32.3444, one month postoperatively = 47.3370, p = 0.0060), and PIF parameters (preoperatively = 0.9196, postoperatively = 1.3419, p = 0.0083). In the entire material of endo-extralaryngeal laterofixation and posteromedial arytenoidectomy with transmuscular posterior cordectomy, mucosal preservation, and laterofixation, there is no significant increase in the FIF50 parameter (preoperatively = 0.5236, one month postoperatively = 0.7468, p = 0.1276) in the early postoperative period, nor a significant decrease in the Rtot parameter (preoperatively = 239.0926, one month postoperatively = 184.1478, p = 0.2399). This can be explained by the fact that a part of our patients had a diagnosis of chronic obstructive pulmonary disease (COPD), so even after the intervention, their Rtot remains high and affects a high SD, and does not lead to significant statistical significance. If we exclude patients with COPD, in further research, we may obtain significance for the second parameter. In the entire material of endo-extralaryngeal laterofixation and posteromedial arytenoidectomy with transmuscular posterior cordectomy, mucosal preservation, and laterofixation, no statistically significant reduction of the Reff parameter can be observed (preoperatively = 160.8962, one month postoperatively = 124.2808, p = 0.1806) in the early postoperative period. This can be explained by the same principle as for Rtot. In future research, if we increase the number of patients and exclude those with COPD, or focus only on younger patients (for example, up to 40 years of age), this parameter may become significant (Table 1).

**Table 1:** Pulmonary function test results before and one month after operative treatment of patients with bilateral paralysis of the vocal folds (both endo-extralaryngeal laterofixation and posteromedial arytenoidectomy with transmuscular posterior cordectomy, mucosal preservation, and laterofixation). FVC: forced vital capacity; FEV1: forced expiratory volume in one second; PEF: peak expiratory flow; PIF: peak inspiratory flow; FIF50: forced inspiratory flow at 50% of FVC; Rtot: total respiratory resistance; Reff: reference values percentage.

	FVC (%)	FEV1 (%)	PEF (%)	PIF (L/s)	FIF50 (L/s)	Rtot (%)	Reff (%)
Preoperative	86.252	56.7741	32.3444	0.9196	0.5236	239.0926	160.8962
1 month after the surgery	93.6174	77.0963	47.3370	1.3419	0.7468	184.1478	124.2808
P-value	0.0286	0.0072	0.0060	0.0083	0.1276	0.2399	0.1806

In our study, 22 patients had endo-extralaryngeal laterofixation as the method of choice for the operative treatment of bilateral paralysis of the vocal folds. Airflow parameters determined by body plethysmography after endo-extralaryngeal laterofixation showed improvement after one month of intervention, with a significant improvement in FEV1 (preoperative = 44.091, one month postoperative = 77.833, p = 0.0252), while the rest of FVC% (preoperative = 80.018, one month postoperative = 85,989, p = 0.0835), PEF% (preoperatively = 34.222, one month postoperatively = 46.716, p = 0.0605), and PIF (preoperatively = 0.6683, postoperatively = 1.1967, p = 0.0626) were at the limit of significance. The significance of values was lost after six months. This can be explained by the medialization of the vocal folds over time. After a month, the thread on the vocal folds cannot be seen because the constant movement of the larynx up and down leads to its ingrowth into the tissue, and thus to the medialization of the vocal folds (Table 2).

**Table 2:** Pulmonary function test results before and after endo-extralaryngeal laterofixation in patients with bilateral vocal fold paralysis. FVC: forced vital capacity; FEV1: forced expiratory volume in one second; PEF: peak expiratory flow; PIF: peak inspiratory flow; FIF50: forced inspiratory flow at 50% of FVC; Rtot: total respiratory resistance; Reff: reference values percentage.

	FVC (%)	FEV1 (%)	PEF (%)	PIF (L/s)	FIF50 (L/s)	Rtot (%)	Reff (%)
Preoperative	80.018	44.091	34.222	0.6683	0.3842	333.93	207.93
1 month after the surgery	85.989	77.833	46.716	1.1967	0.7300	179.87	117.43
P-value	0.0835	0.0252	0.0605	0.0626	0.1864	0.1868	0.1473
6 months after the surgery	89.014	79.714	45.825	1.1340	0.2760	291.72	217.90
P-value	0.8743	0.2788	0.0873	0.2212	0.6112	0.8233	0.7490

In our study, 24 patients had posteromedial arytenoidectomy with transmuscular posterior cordectomy, mucosal preservation, and laterofixation as the method of choice for the operative treatment of bilateral vocal fold paralysis. Airflow parameters determined by pulmonary function test after the surgery showed a statistically significant improvement in PEF (preoperatively = 37.686, one month postoperatively = 47.833, p = 0.0249) and PIF (preoperatively = 0.7900, one month postoperatively = 1.4664, p = 0.0476) one month after surgery. Other parameters were at lower values ​​and indicated a decrease in airway resistance. At six months, the scarring process at the glottis level led to even better airflow values ​​with a significant drop in resistance, and a statistically significant difference in FEV1 (p = 0.0134), PEF (p = 0.0182), and PIF (p = 0.0173) (Table 3).

**Table 3:** Pulmonary function test results before and after the posteromedial arytenoidectomy with transmuscular posterior cordectomy, mucosal preservation, and laterofixation in patients with bilateral vocal fold paralysis. FVC: forced vital capacity; FEV1: forced expiratory volume in one second; PEF: peak expiratory flow; PIF: peak inspiratory flow; FIF50: forced inspiratory flow at 50% of FVC; Rtot: total respiratory resistance; Reff: reference values percentage.

	FVC (%)	FEV1 (%)	PEF (%)	PIF (L/s)	FIF50 (L/s)	Rtot (%)	Reff (%)
Preoperative	84.87	64.643	37.686	0.7900	0.6523	264.31	159.67
1 month after the surgery	93.188	77.428	47.833	1.4664	0.7623	224.77	156.75
P-value	0.2069	0.1368	0.0249	0.0476	0.3035	0.7314	0.9052
6 months after the surgery	91.671	84.250	55.233	1.9820	0.6900	147.78	117.58
P-value	0.1004	0.0134	0.0182	0.0173	-	0.0069	0.0951

In the preoperative assessment of breathing, the majority of patients described their condition as suffocation or shortness of breath that becomes more difficult with physical activity. In another case, the performance of housework was also reduced due to pronounced fatigue. On the visual analog scale of assessment of own breathing, the condition differed after posteromedial arytenoidectomy with transmuscular posterior cordectomy, mucosal preservation, and laterofixation, and after endo-extralaryngeal laterofixation, a significantly better subjective assessment of breathing was observed after the intervention than before the intervention (Table 4).

**Table 4:** Visual analog scale of breathing at rest and during exertion in patients preoperatively and postoperatively.

	Breathing at rest before intervention	Breathing during exertion before intervention	Breathing at rest after intervention	Breathing during exertion after intervention
Endo-extralaryngeal laterofixation	3.2	1.5	8.7	5.3
Submucosal arytenoidectomy	3 (2-5)	1.3 (1-3)	8.4 (6-10)	6.7 (5-10)

## Discussion

Positioning of the vocal folds in a paramedian or median position in cases of bilateral recurrent laryngeal nerve injury provides conditions for relatively preserved phonation with minimal changes in voice quality, but results in significant respiratory difficulties, which vary in severity among patients. The glottic gap during respiration typically ranges from a slit to a maximum of 2 mm in most cases. This space is insufficient for adequate ventilation, and even minimal physical exertion leads to pronounced breathing difficulties.

In our study, we observed that the vast majority of patients (89.1%) had previous thyroid surgery as the cause of bilateral vocal fold paralysis, while significantly fewer patients had another neck surgery or attempted suicide by slaughter. A study conducted by Wiegand et al. recorded that bilateral paralysis of the vocal folds was caused by thyroid surgery in 62.8%, thyroid malignancies in 17.1%, laryngeal malignancies in 8.6%, and trauma in 5.7%, while other causes were less common [6]. This confirms and singles out precisely the iatrogenic bilateral injury of the recurrent laryngeal nerve during thyroid surgery as the dominant cause.

According to our clinical experience, patients often do not present to an otorhinolaryngologist in the early postoperative period. In many countries, thyroid surgeries are performed by general surgeons, and in cases where there is a lack of awareness, patients are not referred to an otorhinolaryngologist. For most surgeons, a “good voice” is considered an indicator that the larynx is intact, even though breathing and phonation are two distinct functional mechanisms of the larynx. Without proper explanation and examination, some patients only seek evaluation and appropriate treatment years later. Having adapted to their condition, with relatively preserved voice quality but significant fatigue during any physical exertion, they often do not realize the underlying issue. Pulmonary hyperinflation, which develops over time, inevitably leads to degradation of the pulmonary parenchyma and creates conditions for pulmonary hypertension. No surgical technique can restore fully mobile vocal folds; rather, it is a balancing act where improving respiratory function inevitably results in deterioration of voice quality. The exact voice outcome after posteromedial arytenoidectomy with transmuscular posterior cordectomy, mucosal preservation, and laterofixation cannot be precisely predicted, but it will inevitably be hoarse and reduced in intensity. In this complex decision-making process, some patients accept surgical intervention in an effort to improve breathing at the expense of voice quality, while a smaller number of patients ultimately refuse surgery.

The objective evaluation of the effects of operative management of bilateral vocal fold paralysis in our study was performed in relation to the findings of pulmonary function tests. Parameters showing the inspiratory quality, i.e., PIF (L/s), FIF50 (L/s), expiratory quality FVC (L), FEV1 (L), and PEF (L/s), and parameters showing airway resistance, i.e., Rtot (kPa*s/L) and Reff (kPa*s/L), were used. Instead of absolute values ​​that show the predicted parameters of the patient in relation to weight and height and values ​​obtained by testing, relative values ​​expressed in percentages were used, except for PIF and FIF50, where the value is expressed in absolute values ​​in L/s. Airflow parameters determined by pulmonary function tests after endo-extralaryngeal laterofixation showed an improvement after one month of intervention, with a significant improvement in FEV1 values ​​(p = 0.0252), while the rest of FVC% (p = 0.0835), PEF% (p = 0.0605), and PIF (p = 0.0626) were at the limit of significance. In a study by Hyodo et al., a statistically significant improvement was noted in the FEV1 parameter (p < 0.01) and a borderline statistically significant improvement in PEF% (p < 0.05) after endo-extralaryngeal laterofixation, which is consistent with our results [8]. Similar results were found in a study published by Leitersdorfer et al., where a statistically significant improvement in PIF (p = 0.01194) and PEF (p = 0.03143) parameters was observed [9]. However, unlike ours and the study conducted by Hyodo et al., no statistical significance was recorded for the FEV1 parameter (p = 0.10036) [8].

The results of our study regarding a statistically significant improvement after posteromedial arytenoidectomy with transmuscular posterior cordectomy, mucosal preservation, and laterofixation in PEF (p = 0.0249) and PIF (p = 0.0476) one month after surgery and in FEV1 (p = 0.0134), PEF (p = 0.0182), and PIF (p = 0.0173) six months after surgery fit with the results obtained in the study by Gamrot-Wrzoł et al., where a statistically significant improvement in PEF (p < 0.001) and FEV1 (p < 0.001) was noted. [10]. However, what we did not find in our study, and what was observed in the study by Gamrot-Wrzoł et al., was a statistically significant improvement in the FVC parameter. Although a statistically significant improvement in the FVC parameter was observed for all operatively treated patients with bilateral vocal fold paralysis, it was not distinguished in the group that underwent posteromedial arytenoidectomy with transmuscular posterior cordectomy, mucosal preservation, and laterofixation. This may be caused by the presence of comorbidity in the form of COPD in our patients who underwent posteromedial arytenoidectomy with transmuscular posterior cordectomy, mucosal preservation, and laterofixation, and not directly by the reduced success of the method itself.

By observing the visual analog scale of the subjective assessment of breathing (1-10) in patients pre- and postoperatively, we recorded a significantly better score postoperatively, just like Gamrot-Wrzoł et al. in their study (p < 0.001) [10]. This proves the success of the described methods with both objective and subjective parameters.

We should not skip or fail to mention new techniques for approaching bilateral recurrent laryngeal nerve paralysis present in the literature, such as reinnervation techniques, laryngeal pacing, gene therapy, and stem cell therapy. In a study conducted by Puxeddu et al., five patients (none of them had a diagnosis of neurological or pulmonary diseases) were treated with bilateral selective laryngeal reinnervation after more than six months without recovery of the recurrent laryngeal nerve bilaterally. Puxeddu et al. found significant correlation between a shorter denervation interval time from surgery and better outcomes, and the spirometry performed 12 months after surgery showed that FVC and FEV1 were not significantly different from the normal reference values in all patients, but overall reduction in the expiratory volumes was detected, as they suppose, due to fixed upper airway alterations in all the patients [11]. As in the study by Puxeddu et al., we also observed a statistically significant improvement in FEV1 parameter after posteromedial arytenoidectomy with transmuscular posterior cordectomy, mucosal preservation, and laterofixation, but without a statistically significant improvement in FVC. Interestingly, our patients had an improvement in both PEF and PIF parameters after surgery. It should also be emphasized that in our study, strict exclusion criteria were not applied as in the previously mentioned study, so some of our patients also had a diagnosis of COPD. Studies have been conducted to examine the usefulness of unilateral and bilateral laryngeal pacing, primarily in animals. Zealear et al. tested the Genesis XP stimulator on canines for bilateral stimulation, and they found that after the procedure, the treadmill test could be completed for canines with bilateral stimulation, unlike for ones with unilateral stimulation [12]. In a review article, Powell et al. reported that the previously used Genesis XP model has been discontinued by St. Jude Medical-Neuro Division, Inc. They also noted that its next-generation system, the Infinity™ deep brain stimulator with eight-channel electrodes and implantable pulse generator (IPG), offers even greater flexibility in channel contact configuration. This system is currently being investigated in their ongoing research, including an FDA-approved clinical trial of bilateral pacing (NCT03085316) [13].

There are several strategies for the future functional recovery of vocal fold movement after severe recurrent laryngeal nerve injury by gene therapy, including targeting axon regeneration enhancement exclusively in the adductor muscle, controlling the expression of neurotrophic factors to improve reinnervation to only the adductor muscles, and reducing misdirected reinnervation between the motor nerve and other fibers in the recurrent laryngeal nerve [14].

Paniello et al. reported that they isolated muscle stem cells from the skeletal muscle of two canines and cultured them, and then implanted them in the posterior cricoarytenoid muscles. They found that glottal resistance and posterior cricoarytenoid muscle strength were increased on the treated side, which is a promising result for the possible application of this technique in the future [15].

The primary limitation of this study, which may have influenced the statistical significance of the results of pulmonary tests, is the fact that patients with COPD were not excluded from this study, so if we exclude COPD patients from the study, we might see the expected statistical significance.

## Conclusions

Lateralization improves inspiration, and most parameters of pulmonary function tests show improvement one month after the intervention, although only one parameter reached statistical significance. Over time, respiratory function deteriorates due to ingrowth of the implanted sutures into the vocal fold tissue, which progressively medializes and narrows the glottic airway. In some patients, the degree of airway narrowing necessitates a second, more permanent intervention.

One month after posteromedial arytenoidectomy with transmuscular posterior cordectomy, mucosal preservation, and laterofixation, respiratory function is improved, and the condition continues to progress positively over time. At six months, the majority of parameters demonstrate statistically significant improvement.
